# Mechanopriming by vascular stiffness and phenotypic reprogramming by disturbed flow: mechanobiology and clinical translation in atherosclerosis

**DOI:** 10.3389/fcvm.2026.1822710

**Published:** 2026-06-24

**Authors:** Jin He, Jun Meng

**Affiliations:** 1 Department of Ultrasound Medicine, The First Affiliated Hospital, Hengyang Medical School, University of South China, Hengyang, China; 2 Department of Function, The First Affiliated Hospital, Hengyang Medical School, University of South China, Hengyang, China

**Keywords:** atherosclerosis, computational fluid dynamics, mechanobiology, mechanopriming, phenotypic reprogramming, wall shear stress

## Abstract

Atherosclerosis exhibits a distinct focal distribution at arterial bifurcations and curvatures, underscoring that systemic risk factors alone are insufficient to fully elucidate its pathogenesis. The coupling of local fluid shear stress—particularly disturbed flow (DF) and oscillatory shear stress (OSS)—with vascular wall stiffness constitutes the core mechanical driver of site-specific plaque progression. This narrative review systematically elucidates the cutting-edge molecular mechanisms of vascular wall-mediated “mechanopriming” and endothelial mechanotransduction. We highlight how the Piezo1 ion channel and the 5-HT1B receptor act as “coincidence detectors,” precisely integrating fluid shear stress and matrix stiffness signals to subsequently activate central signaling hubs such as YAP and c-REL. The dysregulation of these mechanopathways not only triggers pathological reprogramming of endothelial cells—including cGAS-STING-mediated deep senescence, NLRP3-driven pyroptosis, and endothelial-to-mesenchymal transition (EndoMT)—but also impairs RBPJ-epigenetically regulated macrophage efferocytosis and drives pathological matrix remodeling by smooth muscle cells and fibroblasts via complex transcellular communication networks. Clinically, the fusion of multimodal imaging with computational fluid dynamics (CFD), alongside emerging ultrafast ultrasound vector flow imaging, has pioneered novel avenues for the high-fidelity *in vivo* quantification of wall shear stress (WSS). Finally, we critically evaluate current research limitations and prospectively discuss frontier shear stress-targeted therapeutic strategies—such as “mechanodrugs,” biomimetic nanodelivery, and hemodynamic stent optimization—proposing a novel precision cardiovascular medicine framework that formally incorporates localized hemodynamic parameters into established clinical risk stratification algorithms like the ASCVD and SCORE2 models.

## 1 Introduction

Atherosclerosis represents the common pathological substrate underlying ischemic stroke and myocardial infarction and remains the predominant driver of cardiovascular mortality worldwide (1). Although this disease is characterized by chronic inflammation and lipid retention, its etiology is conventionally delineated in terms of systemic risk factors—ranging from diabetes mellitus and hypertension to lifestyle factors such as smoking (1). However, this systemic perspective embodies a fundamental paradox. Despite the widespread impact of hyperlipidemia and metabolic disturbances on the entire vascular system, atherosclerotic plaques do not develop uniformly; rather, they exhibit a strictly localized spatial distribution pattern, preferentially localizing at specific geometric sites such as arterial bifurcations and curved regions (2). This distinctive spatial distribution pattern suggests that abnormalities in blood chemistry alone are insufficient to explain the pathogenesis of the disease. The decisive factor is instead constituted by local hemodynamic forces—particularly fluid shear stress—which serves as a critical “gatekeeping” determinant that translates mechanical forces exerted on the endothelium into site-specific disease progression (3). Underlying this spatial distribution pattern is the differential response of endothelial cells to distinct blood flow patterns, a phenomenon increasingly conceptualized as “flow-mediated endothelial reprogramming” (1). Under physiological conditions, the vascular endothelium is continuously exposed to frictional forces generated by blood flow, namely shear stress. Among these, laminar shear stress (LSS) in straight arterial segments functions as a critical atheroprotective factor that maintains endothelial quiescence by upregulating protective molecules such as endothelial nitric oxide synthase (eNOS) and the transcription factors KLF2 and KLF4, thereby exerting anti-inflammatory, antioxidant, and antithrombotic effects (3). In contrast, at geometrically complex regions such as bifurcations and curved segments, the flow pattern undergoes substantial alteration. disturbed flow (DF), characterized by low magnitude and bidirectional oscillations—designated as oscillatory shear stress (OSS)—overrides these protective signals (2). This aberrant mechanical environment drives a fundamental phenotypic transition in endothelial cells through multiple signaling pathways. Following the initial compromise of barrier integrity, endothelial cells embark upon a maladaptive pathological trajectory manifested by three principal mechanisms: with respect to senescence, OSS induces endothelial cell senescence by blocking autophagic flux (4); with respect to cell death, low shear stress activates the NLRP3 inflammasome and induces endothelial pyroptosis (5); and with respect to phenotypic transformation, OSS drives endothelial-to-mesenchymal transition via the Notch1/p38 MAPK-NF-*κ*B signaling axis, conferring a pro-inflammatory mesenchymal phenotype that accelerates adverse vascular remodeling and atherosclerotic progression (2).

From a clinical perspective, the aforementioned hemodynamic mechanisms provide a solid mechanical foundation for the evolving plaque hypothesis of atherosclerosis (6). Contemporary evidence increasingly challenges the traditional “stenosis hypothesis,” which equates clinical risk simplistically with the degree of luminal narrowing (7). Stone et al. have explicitly articulated that major adverse cardiovascular events (MACE) are associated not solely with severe coronary obstruction but rather with the overall plaque burden across the entire coronary tree (6). In patients following coronary intervention, total plaque volume and percent total plaque burden, as measured by coronary computed tomography angiography (CCTA), are independent predictors of MACE (8). A systematic review and meta-analysis by Gallone et al. further confirmed that high-risk plaque characteristics significantly predict future MACE risk at both the lesion level and the patient level (9). Notably, approximately two-thirds of MACE are not attributable to flow-limiting stenoses but are precipitated by the destabilization of non-obstructive lesions, revealing a profound disconnect between the degree of anatomical stenosis and clinical events (7). The true drivers of plaque prognosis are the biological vulnerability of the lesion and the cumulative overall plaque burden (7). Jukema et al. demonstrated that plaque burden in proximal arteries is the strongest predictor of MACE, an effect that is independent of traditional cardiovascular risk factors (10). Critically, local hemodynamic forces—particularly low shear stress—play a pivotal role in this process. The review by Bacigalupi et al. systematically delineated the mechanisms by which low shear stress and OSS promote plaque growth and pathological remodeling, whereas high shear stress drives vulnerability transformations in obstructive plaques (6). A meta-analysis by Candreva et al. quantitatively confirmed that lower trans-lesional shear stress values are significantly associated with reduced luminal area, increased plaque burden, and enlarged necrotic cores (9). A review by Zhou et al. further noted that while low shear stress promotes atherogenesis, high shear stress regions contribute to the formation of vulnerable plaque phenotypes as lesions progress (11). Additionally, low shear stress can induce the generation of neutrophil extracellular traps (NETs) through Piezo1-mediated mechanosignaling pathways, thereby directly exacerbating atherosclerotic progression (12). Based on these insights, this review aims to integrate biomechanical principles with vascular pathology to systematically elucidate the hemodynamic origins of atherosclerosis. This narrative review not only provides a general overview but also delves into the specific molecular mechanisms by which DF compromises endothelial function—a process increasingly conceptualized as “flow-mediated endothelial reprogramming” (FIRE). Methodologically, the fusion of computational fluid dynamics (CFD) with multimodal imaging technologies has opened new avenues for the clinical quantification of shear stress. Bacigalupi et al. highlighted the significant clinical utility of CFD in understanding stent-vessel interactions, the complexity of flow at coronary bifurcations, and the assessment of lesion severity (6). Research by Gu et al. demonstrated that hemodynamic parameters derived from deep learning-based CCTA are significantly correlated with plaque morphology, and integrating these parameters enhances the predictive accuracy for MACE (7). De Nisco et al. discovered that the combination of high topological shear variation index (TSVI) with lipid-rich plaques exposed to low shear stress is associated with significant plaque progression over a one-year follow-up period, suggesting TSVI as a potential novel biomechanical predictor. Finally, by critically evaluating emerging imaging techniques for shear stress quantification and therapeutic strategies targeting mechanosensitive signaling pathways, this review seeks to bridge the gap between basic research and clinical practice. Regarding therapeutic targets, Piezo1 has been identified as a critical mechanosensitive ion channel responsive to shear stress, playing a central role in OSS-induced endothelial inflammation and representing a promising therapeutic target. Recent comprehensive reviews have systematically summarized the mechanosensitive genes in vascular endothelial cells that respond to blood flow (such as KLF2/KLF4, YAP/TAZ, and NF-*κ*B) and their molecular mechanisms, indicating that targeting these pathways may provide theoretical guidance for the discovery of novel therapeutic strategies aimed at delaying or reversing the progression of atherosclerosis (7).

## 2 Hemodynamic shear stress: classification, multimodal measurement, and mechanobiological background

### 2.1 Biomechanical definition: LSS and DF

From a biomechanical perspective, wall shear stress (WSS) is defined as the tangential frictional force exerted by blood flow on the vascular endothelial surface. It is mathematically expressed as WSS = *μ*·(du/dy), where μ represents blood viscosity and du/dy denotes the velocity gradient perpendicular to the vessel wall (13). Although WSS is typically quantified in Pascals (Pa, where 1 Pa = 10 dyn/cm^2^) or dyn/cm^2^, its biological effects are predominantly dictated by the local vascular geometry (14).

In the straight segments of the arterial tree, blood flow exhibits a unidirectional, high-magnitude laminar flow profile, referred to as LSS, with a physiological value of approximately 15 dyn/cm^2^ (1.5 Pa) (15). LSS acts as a principal guardian of vascular homeostasis by actively maintaining a quiescent, anti-inflammatory endothelial phenotype through the induction of a suite of atheroprotective transcription factors, notably Krüppel-like factor 2 (KLF2) and nuclear factor erythroid 2-related factor 2 (Nrf2) (16). Specifically, LSS upregulates protective molecules such as endothelial nitric oxide synthase (eNOS), KLF2, and Nrf2, thereby preserving the anti-atherosclerotic function of the endothelium (16). Conversely, at arterial bifurcations and curved regions, geometric irregularities disrupt the protective laminar flow profile, leading to flow separation and the emergence of a chaotic hemodynamic environment known as DF (13). In these regions, endothelial cells are exposed to OSS, characterized by low magnitude and bidirectional fluctuations (13). Studies have shown that WSS in OSS regions typically decreases to approximately ±4 dyn/cm^2^ (±0.4 Pa), significantly below the physiological threshold (17). Consequently, this hemodynamic environment fosters the formation of stagnation zones, promoting the local accumulation of pro-atherogenic factors (18). Notably, both low WSS (typically <0.4 Pa) and a high oscillatory shear index (OSI >0.2) are hallmark features of atherosclerosis-prone regions (14).

### 2.2 Multiphysics background: integration of WSS and vascular stiffness

It is worth emphasizing that wall shear stress does not act on vascular endothelial cells in isolation (19). The *in vivo* mechanical environment is far more complex than a single tangential force—endothelial cells, acting as sophisticated integrators, must simultaneously process the tangential dragging force of blood flow shear stress and the circumferential strain generated by pulsatile blood pressure (19). Indeed, in atherosclerosis-prone regions of the arterial circulation, these two mechanical loads are often applied simultaneously (20). Moreover, this hemodynamic interaction is further complicated by the solid mechanical properties of the vessel wall itself: endothelial mechanotransduction depends not only on hemodynamic forces but is also profoundly influenced by the physical properties of the underlying substrate—particularly vascular stiffness (19).

Notably, vascular stiffness increases with age, serving as a hallmark of aging and hypertension (21). Evidence indicates that age-related arterial stiffening—with matrix stiffness increasing from 2.5 kPa to 10 kPa—induces increased endothelial permeability and leukocyte transmigration, both of which are characteristic features of atherosclerosis progression (21). At the molecular level, LSS induces protective signaling through pathways such as KLF2/4, AMPK, and SIRT1, promoting mitochondrial biogenesis and nitric oxide production, whereas OSS enhances glycolysis and inflammation via activation of HIF-1*α* and YAP/TAZ (3). The aging process progressively leads to arterial stiffening and disrupts endothelial homeostasis by shifting these shear-responsive networks from an atheroprotective to an atheroprone state (3). More importantly, *in vitro* model studies have revealed signaling crosstalk between vascular stiffness and fluid shear stress: endothelial cells cultured on more compliant substrates exhibit a more rapid response to LSS, characterized by enhanced endothelial nitric oxide synthase (eNOS) phosphorylation and increased nitric oxide production, thereby amplifying the atheroprotective signals induced by fluid shear stress (19). Conversely, under high shear stress conditions, increased matrix stiffness potentiates the activation of inflammatory signaling pathways in endothelial cells, further exacerbating the pathological response to DF (22).

**Figure 1:**
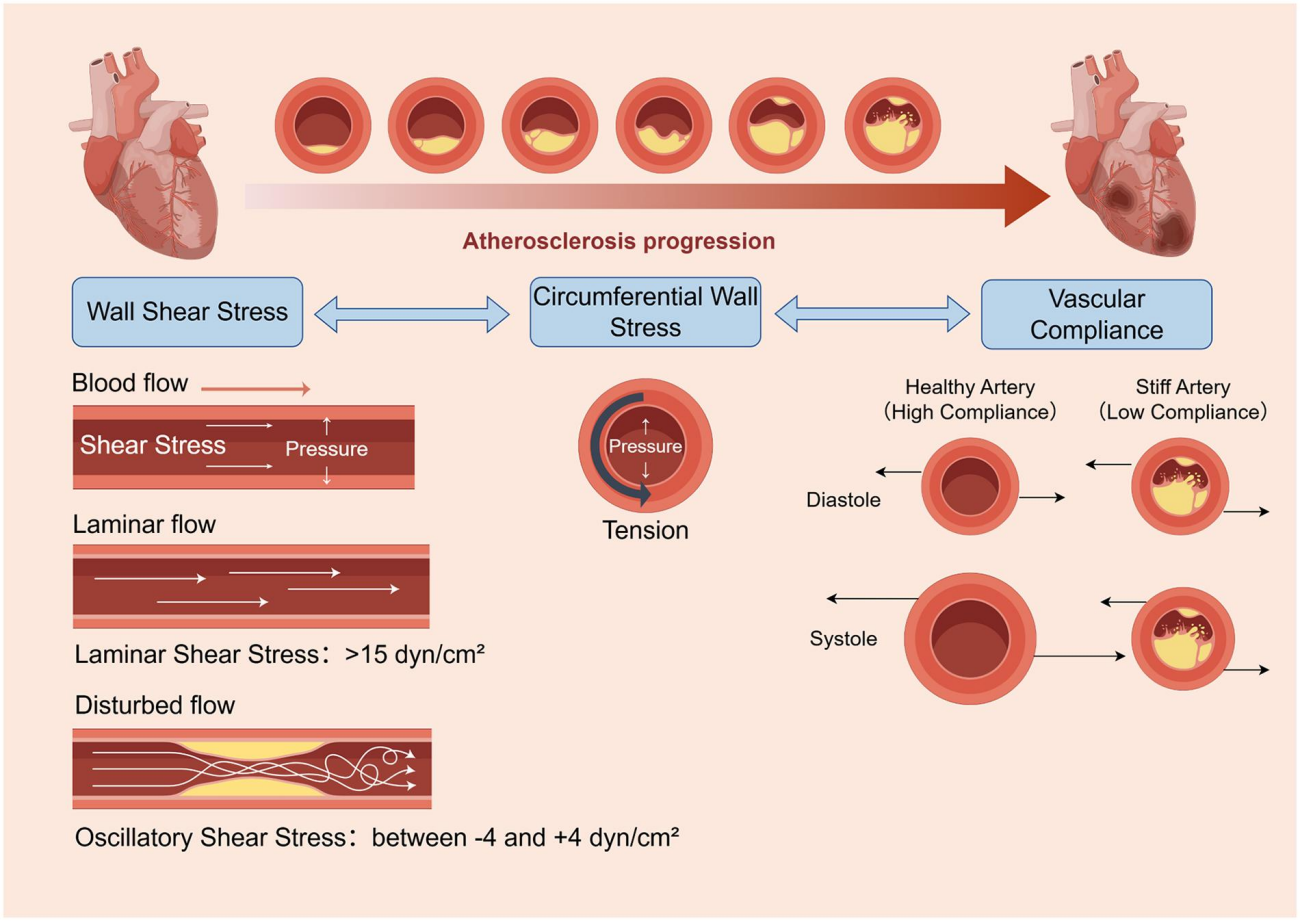
Integrated biomechanical forces driving atherosclerosis. Pathological progression arises from the dynamic crosstalk between wall shear stress (WSS), Circumferential Wall Stress (MWS), and Vascular Compliance. **Left:** WSS profiles contrast atheroprotective Laminar Shear Stress (LSS, unidirectional, >15 dyn/cm^2^) with atherogenic Oscillatory Shear Stress (OSS, bidirectional, ±4 dyn/cm^2^) found at disturbed flow regions. **Center:** MWS represents the perpendicular load generated by pulsatile blood pressure. **Right:** Vascular compliance dictates the magnitude of cyclic strain. Unlike healthy arteries, stiff vessels (low compliance) fail to undergo significant systolic expansion, creating a high-stress, low-strain environment that exacerbates endothelial dysfunction.

In summary, the mechanical microenvironment of endothelial cells constitutes a coupled system comprising multiple mechanical cues, including substrate stiffness and fluid shear stress (19). The capacity of endothelial cells to integrate these multiphysical signals is critical for maintaining vascular homeostasis (19). This integrated multiphysics perspective provides a more comprehensive mechanistic framework for understanding the focal distribution of atherosclerosis (19) (Figure 1).

### 2.3 Multimodal quantification: integration of anatomy and fluid dynamics

Given that these hemodynamic forces are invisible to conventional medical imaging scans, accurate quantification necessitates a hybrid approach (23). In both clinical trials and bench experiments, a promising current strategy for hemodynamic assessment advocates the fusion of the anatomical precision of diagnostic imaging with the predictive capabilities of CFD (3). CFD serves as the computational engine that transforms static anatomical data into dynamic physiological information (23). By ingesting three-dimensional geometric data from optical coherence tomography (OCT), intravascular ultrasound (IVUS), or coronary computed tomography angiography (CTA), this approach effectively constructs a digital twin of the patient's vasculature (23). Within this virtual environment, the application of rigorous boundary conditions—such as specified inlet velocities and blood viscosity—enables us to interrogate the hemodynamic forces acting on the vessel wall (23). However, the fidelity of this digital twin is heavily dependent on these input assumptions; the current lack of standardized protocols for setting boundary conditions remains a significant source of variability (23). The end result is the generation of a high-fidelity, spatiotemporally resolved map of “invisible forces,” pinpointing critical metrics such as WSS and the oscillatory shear index (OSI) that conventional imaging fails to capture (23).

High-resolution intravascular imaging modalities—particularly IVUS and OCT—are indispensable for capturing the detailed morphological structure of the vessel wall (24). These modalities transcend simple visualization; they provide the high-fidelity anatomical constraints required to anchor CFD simulations in reality (24). By explicitly coupling these morphological datasets with fluid dynamics, it becomes possible to dissect the interplay between plaque composition and shear stress (24). For instance, a study by Hartman et al. utilizing a fusion of near-infrared spectroscopy-IVUS and OCT elegantly demonstrated this synergy, revealing that in non-calcified (lipid-rich) segments, low WSS and the presence of lipids exerted a synergistic effect on plaque growth, resulting in the highest plaque progression rate over a one-year follow-up in lipid-rich regions exposed to low shear stress (24).

To overcome the invasive limitations of catheter-based methods, four-dimensional flow magnetic resonance imaging (4D flow MRI) offers comprehensive volumetric assessment by capturing velocity fields in three-dimensional space over time (14). This capability allows for the direct, *in vivo* derivation of WSS distributions without the need for idealized boundary assumptions (25). Woo et al. (published in the *Journal of the American Heart Association*) highlighted the predictive utility of this modality in the context of intracranial atherosclerotic disease (26). Their analysis revealed a critical hemodynamic divergence: while low WSS and high OSI at the inner wall side of curved M1 segments were associated with plaque formation, significant differences in WSS and OSI distribution at curved segments were observed between stroke and non-stroke sides—the stroke side exhibited decreased inner-wall WSS and elevated outer-wall OSI—underscoring the potential contribution of local WSS and OSI variations to stroke risk (26).

### 2.4 Mechanisms of mechanical pre-activation

Ultimately, the mechanical etiology of atherosclerosis cannot be attributed to shear stress alone (27). Rather, the pathology arises from dynamic crosstalk among blood flow shear stress, circumferential strain, and vascular compliance (28). This interplay is nonlinear and synergistic: arterial stiffness not only coexists with shear stress but also functionally calibrates the mechanosensitivity of the endothelium (29).

From a biomechanical perspective, the stiffness of the vascular matrix dictates the basal cytoskeletal tension of endothelial cells (30). In compliant (soft) vessels, the endothelium exists in a state of mechanical quiescence, enabling it to effectively buffer hemodynamic fluctuations (29). However, significant increases in vascular stiffness—hallmarks of aging and hypertension—mechanically prime the endothelium, placing it under high basal tension by enhancing focal adhesion maturation and stress fiber assembly (3). This engenders a “mechanical pre-activation” effect: a stiff extracellular matrix (ECM) lowers the activation threshold of mechanoreceptors (31). Studies have demonstrated that endothelial cells cultured on stiff substrates exhibit fundamentally altered responses to shear stress: when exposed to identical high shear stress levels, cells on stiff matrices display a greater number of differentially expressed genes and enhanced activation of inflammatory signaling pathways (32). The core molecular mechanism underlying this mechanical pre-activation involves mechanosensitive transcriptional coactivators such as YAP/TAZ—stiff substrates promote YAP nuclear translocation, and this pathological signaling is further amplified when DF is superimposed (33).

Consequently, even normally protective levels of LSS may be misinterpreted by tension-primed endothelium; conversely, the pro-inflammatory signals of DF are exponentially amplified when superimposed on a stiff substrate (32). Decoupling this synergistic effect—the “pincer movement” formed by vascular wall strain (circumferential strain) and external fluid forces (shear stress)—is critical for understanding plaque instability (34).

## 3 Endothelial signal transduction and biological effects mediated by shear stress

**Figure 2:**
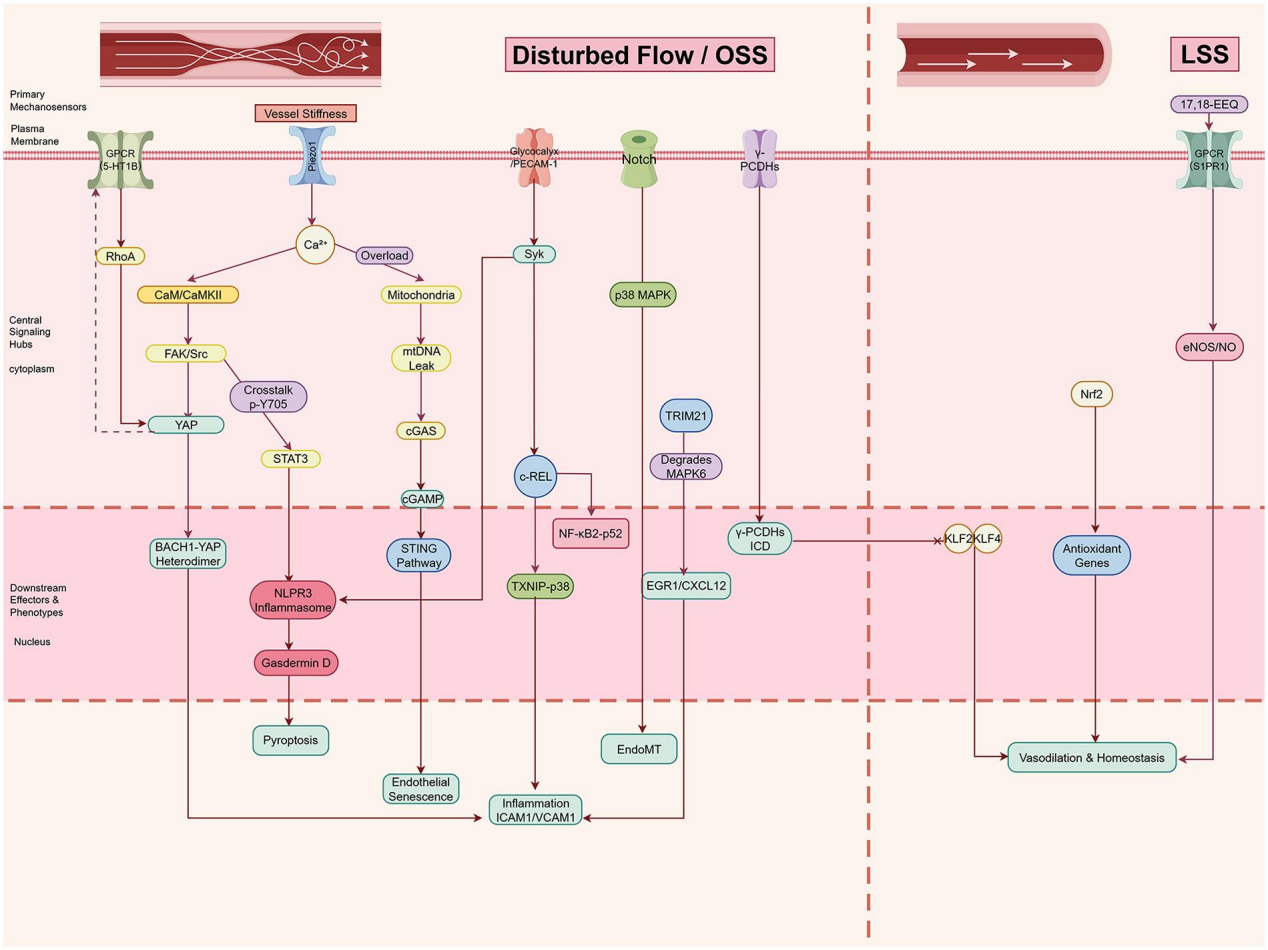
Hierarchical mechanotransduction signaling in endothelial cells. This schematic delineates the Sensors-Hubs-Effectors architecture dictating endothelial fate. **Left:** Disturbed Flow (DF/OSS): Combined shear stress and vessel stiffness activate primary sensors, including Piezo1, 5-HT1B, Notch, and the Glycocalyx/PECAM-1 complex. Notably, a positive feedback loop between 5-HT1B and YAP further amplifies the mechanotransduction. Signals converge on cytoplasmic hubs (Src, Syk, p38 MAPK, c-REL) and YAP, triggering four key pathogenic cascades: (1) Piezo1-Ca^2^⁺-driven mitochondrial DNA leakage and cGAS-STING activation (Senescence); (2) NLRP3 inflammasome-mediated pyroptosis; (3) BACH1-YAP-induced inflammation; and (4) Notch/p38 MAPK-driven Endothelial-to-Mesenchymal Transition (EndoMT). Concurrently, nuclear translocation of *γ*-PCDHs actively suppresses the protective KLF2/4 pathway. **Right:** laminar shear stress (LSS): Physiological flow maintains homeostasis via the S1PR1 receptor (mimicked by 17,18-EEQ) and the Nrf2/KLF2/4 transcriptional axes, promoting vasodilation and antioxidant defense.

The essence of this process lies in the mechanism of mechanotransduction, wherein endothelial cells act as biological translators, converting the physical force of blood flow shear stress into biochemical signals (35). The mechanotransduction of shear stress underpins a variety of cardiovascular benefits, including some of those associated with increased physical activity (35). To deconstruct the complex signaling networks driving atherosclerosis, we employ a hierarchical framework (Figure 2): signaling is initiated by primary mechanosensors, integrated and amplified by central signaling hubs, and ultimately converges on downstream effectors that dictate the atherosclerotic phenotype (13).

Endothelial cells are equipped with a diverse array of mechanosensors to perceive the frictional force of shear stress (35). These sensors include junctional proteins such as platelet endothelial cell adhesion molecule-1 (PECAM-1), the extracellular glycocalyx and its components, ion channels like PIEZO1, as well as caveolae and primary cilia (13). Initial mechanical signals are subsequently transmitted via adaptor proteins and kinases, which function as central signaling hubs responsible for signal integration and amplification (35). Key signaling pathways involve the activation of integrins, particularly *β*1 and *α*v integrins, which are crucial for initiating downstream cascades such as the NF-*κ*B pathway (36). Notably, disturbed flow promotes the activation of YAP/TAZ, thereby enhancing glycolysis and inflammation, whereas atheroprotective LSS suppresses their activity (13). Another critical hub is the transcription factor KLF2, which is strongly induced by atheroprotective shear stress and drives the expression of anti-inflammatory and antithrombotic genes (37). Ultimately, these integrated signals regulate a suite of downstream effector mechanisms that determine endothelial cell phenotype and fate (38). For instance, atheroprotective LSS promotes the expression of eNOS and KLF2, collectively maintaining vascular homeostasis (38). In contrast, atheroprone low shear stress or OSS drives pro-inflammatory signals via the NF-*κ*B pathway (39). Furthermore, OSS significantly promotes endothelial-to-mesenchymal transition (EndMT), a process by which endothelial cells lose their characteristic identity and acquire a pro-fibrotic, migratory phenotype, thereby directly contributing to plaque formation (40).

As detailed in recent authoritative reviews published in *Physiological Reviews*, the vascular endothelium is equipped with an exceptionally vast and diverse array of mechanosensors, encompassing transmembrane structures such as the transient receptor potential (TRP) channel family, various G protein-coupled receptors (GPCRs), integrin complexes, intercellular junctional proteins, and primary cilia (41). However, within the vast mechanotransduction network, this review does not aim to catalog all known mechanosensors but strategically focuses on the Piezo1 ion channel, the 5-HT1B receptor, and the PECAM-1/glycocalyx complex (13). This selection does not imply the insignificance of other sensors; rather, it reflects a critical paradigm shift in vascular mechanobiology: these specific molecules function not merely as fluid shear stress sensors but as coincidence detectors for multi-physical signals (13). In the true pathological microenvironment of atherosclerosis, endothelial cells often face a “two-hit” challenge from both DF and vessel wall stiffening (13). Molecules such as Piezo1 and 5-HT1B are highlighted because recent evidence demonstrates their capacity to integrate two independent mechanical cues—fluid shear stress and matrix stiffness—and to amplify pathological signals through central hubs like YAP (13). Therefore, an in-depth exploration of these sensors with “dual mechanosensing” capabilities holds the most direct clinical and pathological significance for understanding the pathogenesis of atherosclerosis, which is strictly localized to regions characterized by specific geometric and mechanical anomalies (13).

### 3.1 Primary mechanosensors: multidimensional sensing apparatus

To precisely decode the complex mechanical language within the hemodynamic microenvironment, endothelial cells deploy a multilayered sensing network composed of diverse mechanosensors, with a sensing range extending from the glycocalyx on the luminal surface to focal adhesions on the basolateral side (35). Within the intercellular junctional complex, PECAM-1 (platelet endothelial cell adhesion molecule-1) serves as a critical node for shear stress transmission and transduction (42). *In vivo* studies have demonstrated that under low shear stress conditions, PECAM-1 undergoes phosphorylation and recruits/activates the tyrosine kinase Syk; this complex then acts as a proximal sensor to further activate the NF-*κ*B pathway, thereby initiating early inflammation (43). Within this mechanosignaling network, the mechanosensitive ion channel Piezo1 has been identified as a key upstream sentry (35). In regions exposed to DF, Piezo1 expression is significantly upregulated and exhibits enhanced sensitivity to OSS. Critically, Piezo1 also functions as a coincidence detector that integrates matrix stiffness cues (44); when vascular stiffening and OSS risks coincide, Piezo1 activity is further potentiated, maximizing the inflammatory response (45). Beyond the aforementioned structures, 5-HT1B has recently been identified as a novel endothelial mechanosensor critically responsive to disturbed flow (46). OSS induced by DF markedly upregulates 5-HT1B expression, which promotes the nuclear translocation of the transcriptional co-activator YAP by recruiting *β*-arrestin and coordinating RhoA signaling (46). Notably, 5-HT1B and YAP constitute a mutually reinforcing positive feedback regulatory loop: YAP drives the upregulation of 5-HT1B expression, while elevated 5-HT1B in turn amplifies YAP transcriptional activity—a mechanism demonstrated to significantly exacerbate plaque formation in ApoE⁻/⁻ mouse models (46).

### 3.2 Central signaling hubs: integration and amplification

Mechanical signals captured by primary mechanosensors are not transmitted downstream in a linear fashion but rather converge upon specific core transcription factors and kinases for functional amplification (47). Among these, Yes-associated protein (YAP) plays a critical role as a central processor of mechanical stress (48). In regions of DF, YAP nuclear translocation is driven by multiple parallel pathways: first, OSS activates Piezo1 channels to mediate Ca^2^⁺ influx, which subsequently activates the CaMKII and FAK/Src cascade, leading to YAP dephosphorylation and nuclear translocation (49); second, OSS-activated Src kinase can directly phosphorylate STAT3, constituting an additional node for YAP-independent inflammatory signal amplification (50). Furthermore, independent of the Piezo1 pathway, the transcription factor BACH1 possesses intrinsic mechanosensing capabilities and can physically associate with YAP to form a highly transcriptionally active heterodimer, co-inducing the transcription of pro-atherogenic genes (51). Parallel to YAP, another critical node is c-REL, which has been identified as an NF-*κ*B family member that responds specifically to DF (52). Acting as a central command node, c-REL employs a dual-track strategy to simultaneously drive pathological progression: on one hand, it initiates inflammatory cascades via the TXNIP-p38 MAPK axis; on the other hand, it drives aberrant endothelial cell proliferation through the non-canonical NF-*κ*B2-p52 pathway (52).

### 3.3 Downstream effectors: execution of pathology

Activation of central signaling hubs ultimately triggers distinct effector mechanisms that execute programs of cellular senescence, inflammatory cell death, and phenotypic transformation. In terms of senescence, a critical crosstalk occurs between plasma membrane mechanical signals and mitochondrial function: OSS induces mitochondrial damage and mitochondrial DNA (mtDNA) leakage in endothelial cells, which is recognized by cytoplasmic cGAS and subsequently activates the STING pathway, driving endothelial cells into a state of deep senescence accompanied by massive secretion of senescence-associated secretory phenotype (SASP) factors. Concurrently, with respect to cell death, low shear stress activates STAT1 via phosphorylation of IKK*ε*, which in turn upregulates and assembles the NLRP3 inflammasome, ultimately triggering highly inflammatory pyroptosis through Gasdermin D cleavage (53). Beyond transcriptional activation, DF also harnesses the ubiquitin-proteasome system to downregulate host defense mechanisms—for instance, by activating the E3 ubiquitin ligase TRIM21 to target and degrade the protective kinase MAPK6, thereby unleashing the EGR1/CXCL12 axis to exacerbate vascular inflammation (54). Finally, at the level of histopathological remodeling, endothelial-to-mesenchymal transition (EndMT) plays a critical role. OSS primarily drives EndMT by activating Notch3 nuclear translocation and the p38 MAPK-NF-*κ*B signaling axis, remodeling the cellular transcriptome and driving endothelial cells to lose their barrier characteristics while acquiring a pro-inflammatory, pro-fibrotic mesenchymal phenotype (55).

### 3.4 Impairment and decompensation of atheroprotective signals

Notably, the pathogenesis of atherosclerosis arises not only from the activation of pro-inflammatory danger signals but also from the systematic failure of protective signaling networks. Under physiological conditions, LSS executes a genomic suppressive program via KLF2 and KLF4, regulating the expression of the majority of anti-inflammatory and antithrombotic protective genes, while simultaneously driving Nrf2 nuclear translocation to establish antioxidant defenses (56). However, DF does not merely result in the loss of protective signals but also involves active molecular “clogging.” Under DF induction, the conserved intracellular domain of *γ*-protocadherins (*γ*-PCDHs) is cleaved and translocates to the nucleus, where it physically associates with the Notch intracellular domain (NICD), thereby forcibly suppressing Notch signaling required for KLF2/4 transcription (56). Intriguingly, pharmacological strategies can actively mimic the protective effects of LSS; for instance, the EPA metabolite 17,18-epoxyeicosatetraenoic acid (17,18-EEQ) allosterically activates the S1PR1-Gq-Ca^2^⁺-eNOS signaling axis, thereby reconstituting endothelial defenses within the pathological flow microenvironment (57).

## 4 Shear stress and atherosclerosis: from dysfunction to pathological remodeling

### 4.1 Endothelial inflammation, barrier dysfunction and biophysical alterations

Endothelial activation and barrier dysfunction are initiating events in the process of DF/OSS-driven atherosclerosis (13). LSS maintains vascular function by upregulating protective proteins such as eNOS and Nrf2, whereas OSS activates pro-inflammatory pathways including NF-*κ*B and ROS, driving early atherosclerotic lesion formation (58). In the early stages of this pathological process, inflammatory infiltration is particularly prominent. DF/OSS upregulates adhesion molecules (VCAM1, ICAM1) via signaling pathways such as Piezo1-YAP/Src, thereby effectively promoting the infiltration of circulating monocytes (13). The mechanosensitive ion channel Piezo1 is especially critical under LSS; its activation promotes NO release and angiogenesis (59). Endothelium-specific knockout of Piezo1 leads to impaired NO synthesis, compromised vasodilation, and hypertension (60). Regarding the specific mechanisms of inflammatory infiltration, studies have revealed a key role for the cell membrane-associated cytoskeletal protein Talin1 (61). Serum Talin1 levels are significantly elevated in patients with coronary artery disease compared to those without coronary artery disease (62). Both TNF-α and OSS can activate Piezo1 to mediate Ca^2^⁺ influx, which subsequently activates Talin1 and modulates YAP to promote inflammatory responses (63). Talin1 knockdown effectively inhibits such induced inflammatory responses, suggesting that Talin1 represents a potential novel therapeutic target (63).

Concurrently, the structural integrity of the endothelial barrier reflects severe impairment of cellular architecture. Li et al. demonstrated that Rictor, the main constituent protein of mTORC2, is essential for maintaining actin filament polarity in the endothelial cortex (64). Low shear stress disrupts actin filament polarity and promotes the pathological translocation of VE-cadherin, a key component of adherens junctions (64). Following Rictor knockdown, both VE-cadherin translocation and stress fiber formation are increased, while low shear stress-induced von Willebrand factor upregulation is suppressed, indicating that Rictor mediates endothelial injury by regulating adherens junctions and von Willebrand factor (64). In addition, DF/OSS disrupts glycocalyx integrity, downregulates tight junction and adherens junction protein expression, increases endothelial permeability, and promotes low-density lipoprotein deposition and oxidation (65). At a deeper level, this barrier dysfunction is also influenced by mechanosensitive super-enhancer-driven transcriptional reprogramming (66). These large clusters of transcriptional enhancers drive robust expression of prothrombotic genes while simultaneously repressing homeostatic programs (66). Through integrative analysis, studies have characterized unidirectional flow-induced anti-oxidant super-enhancers as well as DF-induced super-enhancers that drive prothrombotic gene expression (66). Recent research has further revealed that the super-enhancer-driven core transcription factor FOXP1 delays endothelial cell senescence via phase separation-mediated SESN3 activation (67). Turning to phase separation, the latest biophysical perspective has uncovered that OSS also disrupts the endothelial barrier by altering the liquid-liquid phase separation properties of proteins. OSS forces hypermethylation of the adaptor protein Kindlin-2 via PRMT5, preventing the formation of liquid-liquid phase separation condensates, thereby effectively dissolving the structural “glue” between cells and significantly increasing the intimal permeability to low-density lipoprotein infiltration (68). Liquid-liquid phase separation of Kindlin-2 in endothelial cells is a critical process for focal adhesion assembly and junction formation. Mass spectrometry analysis revealed that OSS increases PRMT5-catalyzed arginine methylation of Kindlin-2 (with the key site being residue R290), inhibiting phase separation and impairing junction maturation (68). The use of PRMT5 inhibitors to alleviate DF-induced vascular barrier disruption suggests that inhibiting Kindlin-2 arginine methylation may represent a potential therapeutic strategy for vascular diseases (68).

### 4.2 Cell senescence, death and clearance defects

Ultimately, the persistent attack of hemodynamic forces culminates in three concurrent failures: accelerated endothelial cell death, premature senescence, and defective waste clearance. These processes collectively drive the evolution from early dysfunction to advanced necrotic core formation at atherosclerotic lesion sites (69). Within this vicious cycle, pyroptosis and the senescence-associated secretory phenotype (SASP) play initial destructive roles. Low shear stress triggers endothelial cell pyroptosis—an inflammatory form of programmed cell death—via the IKK*ε*-STAT1-NLRP3 signaling axis (53). Mechanistically, low shear stress promotes the phosphorylation of IKK*ε*, which in turn promotes NLRP3 expression through the activation of signal transducer and activator of transcription 1 (STAT1) (53). The activated NLRP3 inflammasome leads to caspase-1 activation, which subsequently cleaves Gasdermin D, forming pores in the cell membrane and ultimately causing membrane rupture and the release of mature IL-1β and IL-18 (53). Meanwhile, surviving cells are forced into a senescent state under persistent mitochondrial stress. In atherosclerosis-prone areas, OSS causes mitochondrial damage in endothelial cells, leading to the leakage of mitochondrial DNA (mtDNA) into the cytoplasm (70). Cytoplasmic mtDNA is recognized by cGAS to produce cGAMP, activating the STING pathway and leading to endothelial senescence, which ultimately results in endothelial dysfunction and atherosclerosis. The abundantly produced SASP factors (including IL-1β, MIP-1*α*, and TNF*α*) further exacerbate local inflammation and induce paracrine senescence in neighboring cells (71). Concomitant with extensive cell death, local waste clearance mechanisms become severely defective. As demonstrated by Wu et al., DF systematically suppresses the expression of MerTK, a member of the TAM receptor family (69). In DF regions, the suppressed expression of MerTK leads to impaired endothelial efferocytosis, resulting in the accumulation of uncleared apoptotic cells (69). Endothelial MerTK deficiency significantly aggravates lesion formation through the upregulation of inflammatory markers and mitochondrial dysfunction (72). Beyond endothelial cells, recent cutting-edge studies have revealed the central role of the transcription factor RBPJ in regulating macrophage efferocytosis (73). As a key transcription factor in the canonical Notch signaling pathway, RBPJ, although not affecting inflammation *per se*, effectively promotes the clearance of apoptotic cells by macrophages within atherosclerotic plaques (73). During efferocytosis, RBPJ upregulates Stard13 and Arsg expression by reducing H3K9me3 levels at their promoter regions; among these, Stard13 enhances actin polymerization through the regulation of Rho and RAC GTPases, thereby effectively promoting macrophage phagocytic capacity (73). Inhibition of Notch signaling reduces efferocytosis, whereas inhibition of SUV39H1/H2, which are responsible for H3K9 trimethylation, amplifies the expression of Stard13 and Arsg and enhances efferocytosis in RBPJ-deficient macrophages (73). In summary, the combination of “pyroptosis generates waste, while dysregulated MerTK and RBPJ signaling prevent waste clearance” constitutes a synergistic pathological cascade, leading to massive accumulation of apoptotic cells and lipid debris. This ultimately culminates in a lipid-rich necrotic core, marking the evolution of atherosclerotic plaques from a stable to a high-risk phenotype.

### 4.3 Mechanosensing and matrix remodeling of smooth muscle cells and fibroblasts

Although endothelial cells serve as the first line of defense in sensing blood flow shear stress, smooth muscle cells (SMCs) in the tunica media and fibroblasts in the tunica adventitia are equally critical participants in mechanotransduction. Their mechanical responses during atherosclerotic progression constitute a deep-seated driving force for pathological vascular remodeling. Under physiological conditions, SMCs primarily bear the cyclic circumferential wall stress generated by pulsatile blood pressure (74). However, when the endothelial barrier is compromised or plaque formation alters local hemodynamics, SMCs become directly exposed to an abnormal microenvironment characterized by interstitial fluid shear stress and increased matrix stiffness. The surface membrane of SMCs is enriched with multiple mechanoreceptors, notably the Piezo1 ion channel and integrin complexes (75). Studies have demonstrated that aberrant physical tension and stiffness drive a phenotypic switch of SMCs from a quiescent contractile state to a proliferative synthetic state through the activation of Piezo1 and the integrin-YAP/TAZ signaling axis (76). Moreover, the recently proposed concept of “smooth muscle cell stiffening syndrome” indicates that, under aging and mechanical stress, SMCs not only secrete excessive matrix but also undergo intrinsic stiffening of their own cytoskeleton (74). This cellular-level stiffening further amplifies aberrant activation of mechanosensitive pathways via a positive feedback mechanism, promoting SMC migration into the intima and participation in fibrous cap formation, and even driving their transformation into foam cells or osteoblast-like cells in advanced stages, thereby exacerbating plaque calcification and vulnerability (76). In addition to SMCs, adventitial fibroblasts, long regarded as merely a supporting component of the vascular wall, are in fact highly mechanosensitive to changes in wall tension and interstitial fluid shear stress (77). Under conditions of local stress concentration induced by plaque expansion or vessel wall stretching due to hypertension, fibroblasts capture ECM physical deformation through their surface mechanosensing apparatus (78). Such mechanical signals, together with pro-inflammatory factors (e.g., TGF-*β*) released within the plaque, strongly induce their differentiation into myofibroblasts, marked by *de novo* expression of *α*-smooth muscle actin (*α*-SMA) and the assembly of highly contractile stress fibers (79). Activated myofibroblasts not only synthesize and rearrange large amounts of collagen, leading to constrictive remodeling, but also release pro-inflammatory factors and matrix metalloproteinases through an “outside-in” paracrine mechanism, further deteriorating the intimal plaque microenvironment (80). Collectively, the mechanosensing mechanisms of SMCs and fibroblasts not only represent a passive adaptation to endothelial injury but also constitute a central hub that actively remodels the lesion microenvironment and dictates plaque stability and pathological evolution.

## 5 Shear stress-based therapeutic strategies

### 5.1 Responsive drug delivery and biomimetics

**Figure 3:**
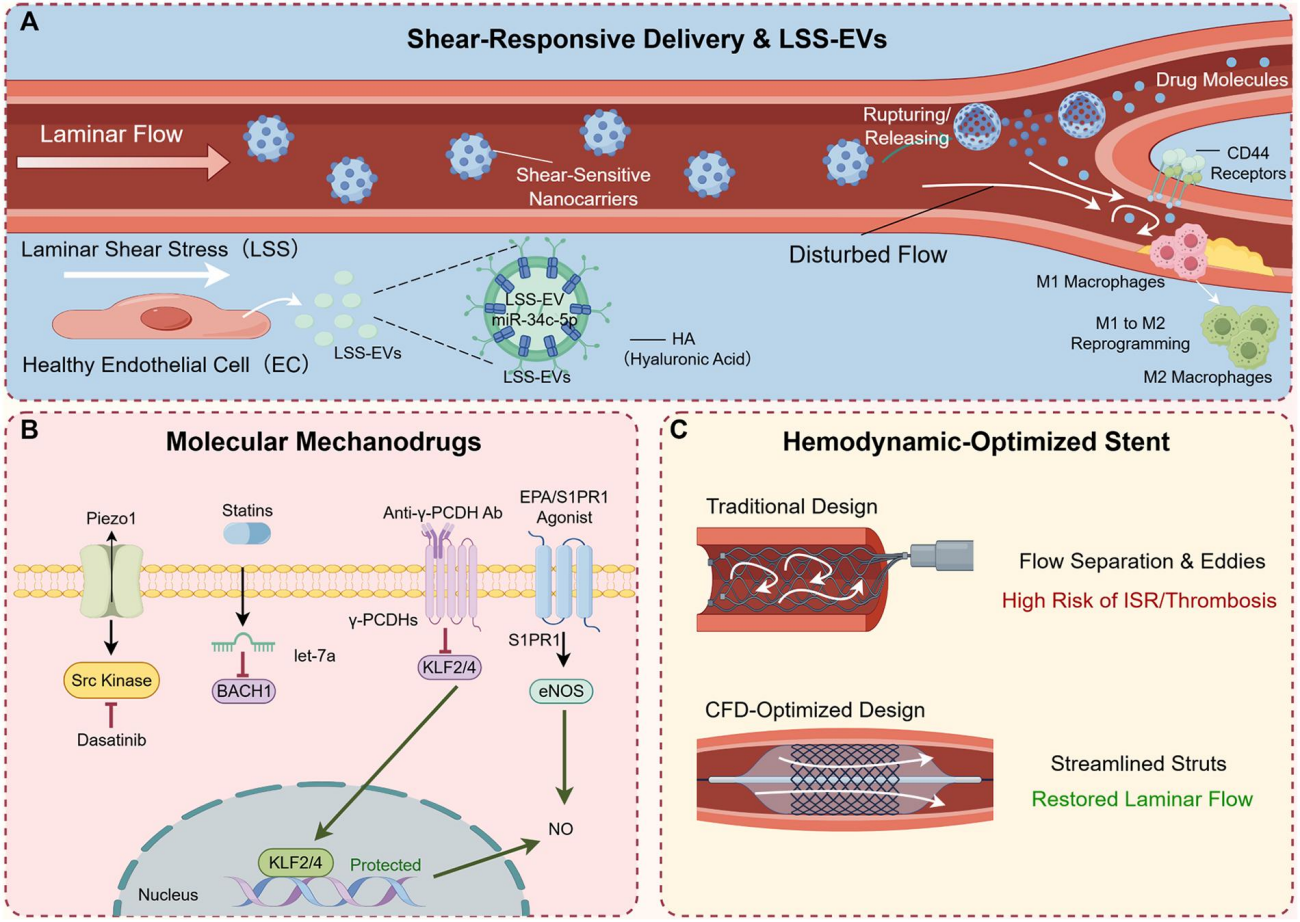
Emerging sheaR,stress based therapeutic strategies in atherosclerosis. **(A)** Shear-Responsive Delivery and Biomimicry. Left: Shear-sensitive nanocarriers (e.g., liposomes) rupture specifically at regions of disturbed flow to release drugs locally. Right: LSS-induced extracellular vesicles (LSS-EVs) containing miR-34c-5p are modified with hyaluronic acid (HA) to target CD44 + macrophages, promoting M1-to-M2 repolarization via the TGF-*β*-Smad3 axis, which further modulates the broader plaque microenvironment via metabolic paracrine signaling. **(B)** Molecular Mechanodrugs. Dasatinib inhibits Piezo1-mediated Src activation to attenuate inflammation. Statins (e.g., Rosuvastatin) suppress the mechanosensor BACH1 via let-7a upregulation. Anti-*γ*-PCDH antibodies restore protective KLF2/4 expression by neutralizing *γ*-protocadherins. EPA and S1PR1 agonists activate the eNOS pathway by mimicking physiological LSS signaling. **(C)** Hemodynamic Stent Optimization. Traditional thick struts (Left) generate flow separation and OSS, promoting In-Stent Restenosis (ISR). CFD-optimized streamlined struts (Right) preserve laminar flow and minimize hemodynamic disturbance.

The marked hemodynamic differences between atherosclerosis-prone regions (disturbed flow/OSS regions) and healthy vascular regions (LSS regions) provide a unique theoretical basis and physical target for the design of intelligent drug delivery systems (15) (Figure 3). Based on this characteristic, researchers have developed shear-sensitive nanocarriers, which are engineered to trigger targeted drug release only under specific hemodynamic conditions (e.g., high shear stress or oscillatory flow), thereby significantly increasing local drug concentration at the lesion site while minimizing systemic side effects (81). In recent years, various strategies have emerged to achieve this goal, including the use of shear-responsive chemical bonds or the construction of supramolecular structures that dissociate under mechanical force, enabling on-demand drug release (81). Moreover, engineering nanoparticles into specific shapes (e.g., discoidal geometry) can optimize their margination and adhesion behavior in disturbed flow regions, thereby improving drug delivery efficiency (82). Beyond synthetic carriers, biomimetic strategies have also demonstrated considerable potential. In a breakthrough study, Li et al. proposed an innovative biomimetic strategy utilizing hyaluronic acid (HA) modification of extracellular vesicles derived from endothelial cells subjected to LSS (LSS-EVs) to target macrophages (15). Mechanistic investigations revealed that these LSS-EVs are enriched in miR-34c-5p, which reprograms pro-inflammatory M1-type macrophages into a reparative M2 phenotype by targeting the TGF-*β*-Smad3 signaling pathway (15). However, when applying such cell reprogramming-based therapeutic strategies, it is imperative to extend the perspective to include the paracrine effects of macrophage lipid metabolism. Indeed, macrophage metabolic reprogramming—particularly alterations in lipid metabolism—not only shapes their own phenotype but also profoundly influences neighboring stromal cells through the secretion of various bioactive molecules (83). Recently, Sadaf et al., employing spatial metabolomics, revealed that cardiac macrophages undergoing metabolic reprogramming significantly upregulate key fatty acid synthesis enzymes, including ATP citrate lyase (ACLY) and fatty acid synthase (FASN) (83). ACLY acetylates the promoter region of the upstream regulator Krt17, driving the production of pro-fibrotic cytokines, including IL-33, which in turn propagates the expansion of pathogenic fibroblasts via a paracrine mechanism (83). This finding unveils a critical transcellular metabolic regulator*y* axis. Although this study primarily focused on post-myocardial infarction cardiac remodeling, its core mechanism is highly likely to operate within the atherosclerotic plaque microenvironment as well. Within the plaque, altered macrophage fatty acid synthesis metabolism may influence the phenotypic switching and ECM remodeling of vascular fibroblasts or smooth muscle cells through similar paracrine signaling axes, thereby contributing to plaque progression and stability regulation. Therefore, when designing LSS-EVs-based anti-atherosclerotic therapeutic strategies, it is essential not only to focus on their direct reprogramming effects on macrophage phenotype but also to systematically evaluate the impact of such interventions on the metabolism and function of downstream stromal cells—particularly the regulatory networks involving the fatty acid synthesis-paracrine axis. Such comprehensive assessment may provide new theoretical foundations and therapeutic targets for the development of more holistic treatment approaches.

### 5.2 Shear stress-based optimization of stent design

Any endovascular interventional procedure inevitably alters the local hemodynamic microenvironment. As highlighted in a 2024 review by El Khoury et al. published in *The Journal of Cardiovascular Surgery*, endovascular treatment of peripheral arteries faces severe challenges, including heavy complex plaque burden, multiple serial stenoses and occlusions, sluggish blood flow, low mean shear stress, and OSS, coupled with repetitive axial, radial, and torsional deformations that significantly hinder the durability of therapeutic outcomes (84). In particular, neo-disturbed flow regions or OSS zones induced by stent struts have been established as primary drivers of in-stent restenosis (85). To address this paradigm, modern stent design has extensively adopted CFD simulation as a core optimization tool (86). Through high-resolution CFD simulations, researchers can evaluate the post-implantation arterial blood flow and shear stress distribution with unprecedented detail. Studies have demonstrated that stents exert a profound impact on the magnitude, direction, and distribution of wall shear stress, and adverse flow perturbations (such as particle accumulation at stent struts and unphysiological shear stress) are highly sensitive to individualized stent geometries (87). Therefore, optimizing strut geometry via computational simulations to minimize flow disturbances—thereby effectively mitigating the risks of restenosis and thrombosis—has emerged as a pivotal strategy in contemporary stent design. Recent literature further emphasizes that multidimensional stent design optimization—encompassing streamlined profiles, reduced strut thickness, and optimized inter-strut spacing—represents the future trajectory for modulating endothelial shear stress; furthermore, the integration of fluid mechanics-based optimization with cutting-edge technologies, such as functional coatings that promote re-endothelialization, is vigorously propelling the clinical translation of next-generation vascular stents (87).

### 5.3 Druggable targets in downstream shear stress pathways (mechanotherapeutics)

If modifying the physical forces themselves proves unfeasible, a highly promising alternative strategy is to block their downstream pathological signal transduction. Initially, investigations into kinase pathways have revealed that OSS promotes the phosphorylation of Src at the Y416 site in endothelial cells via the mechanosensitive channel Piezo1, subsequently activating the Stat3 signaling pathway to drive inflammation (88). Pharmacological inhibition of Src (e.g., via dasatinib) or silencing Src through siRNA effectively abolishes OSS-induced endothelial inflammation; notably, the oral administration of dasatinib in murine models has been shown to significantly attenuate plaque formation (88). Beyond direct kinase targeting, modulating mechanosensitive transcription factors also exhibits substantial therapeutic potential. For instance, BACH1 has been identified as a mechanosensor for hemodynamic stress, forming a transcriptional complex with YAP under OSS stimulation to promote the expression of pro-inflammatory factors (89). Experimental evidence confirms that rosuvastatin can inhibit BACH1 expression by upregulating endothelial microRNA let-7a, thereby exerting a potent anti-inflammatory effect independent of its traditional lipid-lowering properties (89).

Conversely, relieving the “molecular blockade” of protective pathways offers a novel interventional paradigm. In atherosclerotic endothelial cells, clustered *γ*-protocadherins (*γ*-PCDHs) undergo cleavage and translocate into the nucleus, where they physically associate with and profoundly suppress the protective Notch-KLF2/4 signaling axis (90). Antibody-mediated blockade or genetic deletion of *γ*-PCDHs can restore the expression of protective genes and mitigate vascular inflammation without compromising host defense mechanisms (90). Alternatively, atheroprotective mechanosignals can be actively mimicked through chemical interventions. For example, 17,18-EEQ, an eicosapentaenoic acid metabolite, can recapitulate the vasoprotective effects of LSS by activating the S1PR1-Gq-Ca^2^⁺-eNOS signaling axis—the definitive pathway through which the prescription drug Vascepa exerts its clinical efficacy (57).

In advancing these therapeutic strategies, the rigorous consideration of target safety is paramount. Interventions directed at endothelium-specific pathways (e.g., *γ*-PCDHs) demonstrate superior systemic safety profiles compared to broad-spectrum kinase inhibitors (such as dasatinib, which may impair immune function). This distinction underscores the inherent advantages of precision therapies that selectively target the localized vascular mechanotransduction network rather than suppressing the systemic immune system.

## 6 A novel perspective on shear stress research: spatial omics and the mechano-transcriptomic atlas

### 6.1 The mechano-transcriptomic atlas framework

To overcome the inherent limitations of conventional single-cell RNA sequencing (scRNA-seq)—specifically, the loss of spatial contextual information during tissue dissociation—we propose the construction of an integrated mechano-transcriptomic atlas framework (91). Emerging technologies, such as spatial transcriptomics, enable the direct *in situ* mapping of flow-induced transcriptional alterations while preserving the spatial heterogeneity of endothelial cells within their native microenvironment (92). Concurrently, patient-specific CFD modeling allows for the precise quantification of WSS distribution within localized hemodynamic environments, predicated on individualized anatomical geometries (93). By digitally fusing patient-specific CFD hemodynamic models with spatial transcriptomic profiles, this strategy effectively bridges the long-standing data chasm between macroscopic fluid dynamics and microscopic molecular biology (93). Ultimately, this integrative approach empowers researchers to directly ascertain whether localized shear stress patterns spatially overlap with specific pathological hotspots, thereby furnishing novel mechanistic insights into shear stress-mediated vascular diseases (91).

### 6.2 Proof-of-concept study in carotid plaques

A milestone proof-of-concept study published by Sun et al. has provided a pivotal paradigm for the application of spatial transcriptomics in atherosclerosis research (94). This investigation integrated histopathology, electron microscopy, bulk RNA sequencing, and spatial transcriptomics to systematically analyze the proximal, most stenotic, and distal regions of human carotid plaques along the longitudinal axis of blood flow; concurrently, genome-wide association studies were employed to evaluate heritability enrichment and causal relationships, while the prognostic relevance of differentially expressed genes (DEGs) to cardiovascular events was validated in an independent cohort (94). The results demonstrated that within human carotid atherosclerotic plaques, ruptures predominantly occurred in the proximal and most stenotic segments rather than the distal regions, a spatial vulnerability corroborated by histopathological and electron microscopic evidence indicating pronounced features of plaque instability and thrombosis in these susceptible zones (94). By charting the molecular landscape of human carotid plaques using spatial transcriptomics, the authors validated, for the first time in human atherosclerosis, specific signaling pathways intimately associated with the rupture-prone proximal regions (94). Notably, matrix metallopeptidase 9 (MMP9) emerged prominently among the top three most significant DEGs, with Mendelian randomization analysis substantiating a causal link between elevated circulating MMP9 levels and an increased risk of atherosclerosis (94). Collectively, these findings elucidate that rupture-associated genetic signatures (e.g., MMP9) are specifically enriched in the proximal shoulder regions subjected to complex hemodynamics, thereby providing compelling evidence that distinct anatomical locations fundamentally govern localized pathological programs (94).

### 6.3 Technical limitations in resolution and registration

Despite the promising prospects of integrating spatial omics with CFD, its practical implementation continues to confront two core technical challenges (95). The primary obstacle is the intrinsic trade-off between spatial resolution and the field of view (95). Current mainstream spatial transcriptomics platforms (e.g., the 10× Genomics Visium platform) exhibit a fundamental dichotomy between achieving high spatial resolution and maintaining broad transcriptomic coverage (95). Specifically, these sequencing-based platforms typically feature capture spots with a diameter of 55 µm and a center-to-center distance of 100 µm, encapsulating the signals of approximately 1 to 10 cells per spot on average (95). Such a multicellular spatial averaging effect risks masking rare, cell-type-specific mechanosensitive events; mechanosensitive transcripts—including specific ion channels or shear stress-responsive genes—are frequently expressed transiently in a restricted cellular subpopulation, rendering their signals highly susceptible to being overshadowed by the background expression of surrounding stroma (96). To circumvent this limitation, ultra-high-resolution platforms such as Stereo-seq (sub-micron resolution) and Visium HD (2 µm resolution) have recently emerged; nevertheless, they still face substantial constraints pertaining to operational costs, tissue coverage area, and the comprehensiveness of gene detection panels (95).

Another formidable challenge lies in the cross-modal 3D-to-2D data registration (97). Accurately projecting the precise WSS vector fields—derived from CFD simulations of the 3D vascular tree—onto the 2D plane of histological tissue sections remains a daunting technical barrier (97). This challenge is compounded by a confluence of factors: tissues inherently undergo nonlinear deformations during sectioning, fixation, and staining procedures; adjacent tissue sections inevitably incur batch effects due to independent sequencing runs while lacking shared spatial coordinates; and the integration necessitates the joint alignment of highly disparate modalities (i.e., continuous CFD simulation data, morphological histological images, and discrete spatial transcriptomic data) within a unified coordinate framework (97). To tackle these complexities, researchers are actively exploring advanced non-rigid registration algorithms. Prominent examples include deep learning-based unsupervised landmark detection methodologies (e.g., Effortless Landmark Detection) and end-to-end computational solutions designed for *de novo* 3D tissue reconstruction, such as STAIR (98). These sophisticated approaches attempt to leverage neural network-guided thin-plate splines or heterogeneous graph attention mechanisms to effectively resolve nonlinear deformations across histological sections, thereby ultimately realizing the high-fidelity fusion of macroscopic fluid dynamics modeling with microscopic spatial sequencing topographies (98).

## 7 Challenges and perspectives 7.1 future challenges: decoding epigenetic memory

Accumulating evidence indicates that disturbed flow not only triggers immediate transcriptional alterations but also induces and sustains a deleterious, pathological epigenetic memory within vascular endothelial cells. At the level of histone methylation, the repressive histone modification mark H3K27me3 is significantly enriched in atherosclerosis-susceptible regions (99). Studies have demonstrated that OSS upregulates the expression of the histone methyltransferase EZH2, thereby augmenting H3K27me3 abundance (99). Chromatin immunoprecipitation (ChIP) analyses have further corroborated that H3K27me3 directly binds to the promoter region of the *CDH5* gene and silences its transcription, leading to the downregulation of VE-cadherin expression and consequently inflicting permanent damage to endothelial barrier integrity (99). Conversely, the knockdown of EZH2 enhances the enrichment of genes associated with cell-matrix and cell-cell adhesion, subsequently bolstering the stability of the endothelial barrier (99).

Meanwhile, at the level of RNA epitranscriptomics, OSS downregulates the expression of the N6-methyladenosine (m6A) methyltransferase METTL3 (100). m6A sequencing and functional studies have revealed that m6A modification accelerates the degradation of the mRNA of the vascular pathophysiological gene *EGFR*, thereby impairing endothelial repair mechanisms (100). Specifically, m6A modification of the 3’ untranslated region (3'UTR) of *EGFR* hastens its mRNA degradation, whereas double mutation of the *EGFR* 3'UTR abolishes METTL3-induced luciferase activity (100). Adenovirus-mediated overexpression of METTL3 significantly attenuates EGFR activation and endothelial dysfunction under OSS conditions; furthermore, thrombospondin-1 (TSP-1), an EGFR ligand, is specifically expressed in atheroprone regions and remains unaffected by METTL3 regulation (100). Confronting these profound molecular imprints, future research must be dedicated to “erasing” this detrimental epigenetic memory. Currently, multiple studies indicate that targeting histone-modifying enzymes (e.g., EZH2 inhibitors) and RNA methylation regulatory mechanisms holds substantial therapeutic potential for restoring vascular homeostasis. Further elucidating the mechanisms underlying the establishment, maintenance, and erasure of endothelial epigenetic memory will provide an indispensable theoretical foundation for the development of novel preventive and therapeutic strategies against atherosclerosis.

### 7.2 Integration mechanisms of multiphysical signals: YAP/TAZ as the convergence hub

A critical frontier lies in deciphering how diverse physical cues—such as wall shear stress, membrane tension, and arterial stiffness—are integrated within endothelial cells and subsequently transduced into specific transcriptional outputs (101). Based on emerging evidence, we herein propose a “mechanopriming hypothesis,” positing that the full activation of YAP/TAZ adheres to a sequential “priming-and-triggering” two-step temporospatial mechanism (102). In this paradigm, vascular stiffness initially serves as the “priming signal” (102). During pathological vascular stiffening, endothelial cells sense the augmented extracellular matrix rigidity via integrin-based focal adhesions, which elevates cytoskeletal tension and consequently primes YAP/TAZ by enhancing their potential for nuclear translocation (101). Subsequently, aberrant flow patterns act as the pathological “trigger”. In atheroprone regions, such as arterial bifurcations, OSS generated by DF activates the mechanosensitive ion channel Piezo1 on the endothelial plasma membrane. This activation elicits intracellular calcium fluctuations and initiates downstream kinase cascades, which in turn impel the pre-primed YAP/TAZ to undergo nuclear translocation, ultimately initiating pro-atherogenic gene expression (13). Within this multidimensional integration model, YAP/TAZ fundamentally functions as an “AND gate”—a core logical hub for mechanotransduction (103). Vascular stiffness (the priming signal) and DF (the trigger signal) do not operate in isolation; rather, they synergize to constitute a “double hit,” whereby only the concurrent presence of both stimuli can maximally force YAP/TAZ into the nucleus (103). This stiffness-flow crosstalk mechanism profoundly implies that targeting the integrated Integrin-Piezo1-YAP signaling axis may potentially “desensitize” endothelial cells to pathological hemodynamic stress, thereby unveiling a novel therapeutic strategy for atherosclerosis intervention (13).

### 7.3 Clinical standardization of multimodal imaging and CFD integration

The clinical adoption of CFD has been substantially impeded by its exorbitant computational costs, technical complexity, and the prevailing lack of standardized protocols. These inherent limitations not only hinder the seamless integration of CFD into large-scale clinical workflows but also restrict the curation of large, annotated hemodynamic datasets requisite for training artificial intelligence (AI) models. Currently, profound heterogeneities in CFD modeling methodologies, boundary condition specifications, and definitions of hemodynamic parameters across disparate studies further obstruct the effective comparison and synthesis of research findings. In recent years, an increasing number of studies have leveraged deep learning architectures as surrogate models for CFD to achieve accelerated flow field predictions. For instance, Sarkhosh et al. (2025) proposed a deep learning model predicated on the U-net architecture to predict time-averaged wall shear stress (TAWSS) at coronary bifurcations; remarkably, this model generated results in under one second, yielding a normalized mean absolute error of merely 2.53% (104). Similarly, Malek et al. (2025) utilized an expansive dataset comprising 6,858 synthetic left coronary artery bifurcation geometries to train 14 machine learning algorithms, among which the decision tree regression model demonstrated preeminent performance in predicting both TAWSS and the oscillatory shear index (OSI) (TAWSS: R^2^ = 0.999, MAE = 0.000587) (105). Additionally, Nannini et al. (2025) benchmarked six geometric deep learning architectures and discovered that Transformer-based frameworks yielded optimal performance when processing patient-specific data characterized by complex and heterogeneous topologies, enabling highly robust predictions of virtual fractional flow reserve (vFFR) fields (106). Future endeavors must concentrate on establishing standardized, high-throughput simulation workflows driven by AI/machine learning technologies. Concurrently, large-scale, prospective clinical trials are imperative to validate the clinical utility of hemodynamic parameters—such as wall shear stress and membrane tension—as superior predictors of MACE compared to conventional anatomical stenosis metrics. Moreover, the integration of multimodal imaging data (e.g., CCTA, CMR, PET-CT) with CFD and deployable AI workflows has demonstrated the remarkable potential to augment ischemia detection capabilities by 28% and lesion classification accuracy by 35%, thereby forging a transformative paradigm for comprehensive cardiovascular evaluation (107).

### 7.4 Emerging ultrasound technologies for non-invasive shear stress assessment

Ultrasound technology is undergoing a profound paradigm shift, progressively evolving from a conventional morphological diagnostic tool into a multiparametric mechanodiagnostic platform that seamlessly integrates structural imaging with hemodynamic mechanobiology assessment. The non-invasive, *in vivo* quantification of WSS has long been a pursuit in cardiovascular biomechanics, and emerging ultrasound technologies are systematically translating this vision into clinical reality. Among these, ultrafast ultrasound vector flow imaging (VFI), predicated on plane-wave transmission and high-frame-rate architecture, has emerged as one of the most clinically promising modalities for *in vivo* WSS assessment, owing to its unique capability to accurately quantify complex blood flow vector fields without the necessity for angle correction. For instance, in the evaluation of carotid atherosclerotic stenosis, high-frame-rate VFI has successfully achieved dual-parameter quantitative analysis of the peri-stenotic turbulence index and WSS; their combined diagnostic performance yielded an area under the curve (AUC) of 0.899, demonstrating superior early-warning potential compared to traditional stenosis grading methodologies (108). Concurrently, a clinical validation study encompassing 47 common carotid arteries further substantiated that high-frame-rate VFI (HiFR-VFI) is highly comparable to phase-contrast magnetic resonance imaging (PC-MRI) in WSS measurements, demonstrating a Pearson correlation coefficient of 0.60 and a mean absolute error of 27.6%, thereby providing a robust foundation for the clinical credibility of ultrasound-derived WSS estimation (109). Furthermore, high-frequency ultrasound has demonstrated unique utility in the *in vivo* assessment of vascular WSS. For example, Kim et al. utilized an 18-MHz linear array transducer to successfully achieve time-varying dynamic mapping of 2D and 3D velocity fields and WSS within the left anterior descending coronary artery of an *ex vivo* pulsatile porcine heart. The root-mean-square error of the 2D velocity magnitude was less than 8% of the maximum velocity recorded by the reference system, establishing a critical technological cornerstone for the future implementation of dynamic intracoronary WSS assessment via forward-viewing intravascular ultrasound devices (110).

In broader clinical applications, VFI has been employed to map WSS, the oscillatory shear index (OSI), and relative residence time across multiple topographical segments of the femoropopliteal artery. It was discovered that the distal superficial femoral artery at the adductor canal exhibited significantly lower WSS and higher OSI, which strongly corroborated the recognized high incidence of atherosclerotic plaques and occlusions in this specific anatomical region. This finding further validates the biological efficacy of ultrasound-derived WSS profiling in elucidating the regional susceptibility to atherosclerosis (111). Regarding the integration of hemodynamics and mechanobiology, recent pioneering work by Xia et al. validated the feasibility of using high-frequency ultrasound to map the spatial distribution of *in vivo* WSS, subsequently establishing a direct predictive linkage between non-invasive WSS data and the activation of the Syk/PECAM-1 signaling axis. The study delineated that low shear stress activates Syk via the PECAM-1 signaling pathway both *in vivo* and *in vitro*, thereby orchestrating inflammatory responses. Notably, the administration of the Syk inhibitor R406 significantly mitigated the low shear stress-induced endothelial inflammation, offering a potential point-of-care observational window into the hemodynamic etiology of atherosclerosis (112). Notably, the synergistic integration of VFI with radiofrequency-signal-based quantitative vascular stiffness analysis (e.g., R-QVS) holds immense promise for simultaneously capturing the mechanical loading environment (WSS) and the structural mechanical properties (stiffness coefficients, pulse wave velocity) of the vascular wall, thereby facilitating an integrated, multidimensional risk assessment for atherosclerosis. Looking ahead, an ongoing bidirectional cohort study involving 855 patients with asymptomatic carotid plaques aims to develop a regression prediction model for MACE by combining the Plaque Reporting and Data System (Plaque-RADS) with VFI. Its findings are anticipated to furnish high-level, evidence-based medical data to substantiate the practical application of ultrasound WSS evaluation in clinical risk stratification. Overall, emerging ultrasound technologies are outfitting clinicians with unprecedented, non-invasive *in vivo* mechanodiagnostic tools to interrogate the hemodynamic origins of atherosclerosis. Despite extant challenges pertaining to technological standardization, large-scale clinical validation, and multiparametric integration, their assimilation into routine cardiovascular diagnostic workflows portends an exceptionally broad and transformative clinical horizon.

### 7.5 Clinical translation and precision medicine

The clinical translation of shear stress-modulating therapeutics is confronted with multiple hurdles. Although therapies based on extracellular vesicles (EVs) hold tremendous promise, their large-scale industrialization remains hampered by the critical bottleneck of low secretion yields, which restricts their widespread clinical application in precision medicine domains such as shear stress modulation. Vertical wheel bioreactors (VWBRs), by cultivating human vascular organoids under precisely controlled shear stress conditions, enable the scalable production of EVs. This approach can augment yields by 2- to 3-fold compared to conventional static cultures, providing a promising technological trajectory to surmount this manufacturing barrier (113). Furthermore, the vascular safety profiles of nanocarriers, such as ultrasmall superparamagnetic iron oxide nanoparticles (USPIONs), warrant rigorous scrutiny. Studies demonstrate that the surface chemistry of nanoparticles significantly dictates their interaction with the protein corona, endothelial cellular uptake, viability, and barrier function; certain functional modifications can even induce the downregulation of endothelial-specific adhesion proteins (VE-cadherin and PECAM-1) and subsequently exacerbate endothelial permeability (114). More concerningly, while the mature endothelial glycocalyx acts as a negatively charged barrier affording protection against cationic nanoparticles, the pathological degradation of this barrier in disease states such as diabetes can drastically alter the uptake dynamics and toxicity profiles of these nanocarriers (115).

Within the framework of precision medicine, any robust risk stratification model must meticulously account for individual biological heterogeneity. Sex differences serve as a crucial regulatory determinant: estrogen exerts vasoprotective effects via the endothelial estrogen receptor alpha (ER*α*). ER*α* not only mediates flow-mediated dilation (FMD) and flow-mediated outward remodeling (FMR) but also acts directly as a membrane-associated mechanosensor in shear stress mechanotransduction cascades. This elucidates the underlying mechanisms explaining why premenopausal women exhibit a markedly lower risk of cardiovascular disease compared to age-matched men (116). Comorbidities, from an alternative dimension, profoundly modulate the sensitivity of the vascular wall to hemodynamic forces. Taking diabetes as a paradigm, chronic hyperglycemia-induced degradation of the endothelial glycocalyx compromises shear stress mechanotransduction, propelling the endothelium from an atheroprotective, anti-inflammatory state to a dysfunctional, injury-susceptible phenotype, thereby accelerating the initiation and progression of atherosclerosis (115).

A truly robust risk model necessitates the superimposition and integration of an individual's hemodynamic profile with their intrinsic biological susceptibility. Specifically, future precision medicine frameworks must explore the formal incorporation of localized abnormal shear stress indices (e.g., low wall shear stress and elevated oscillatory shear index) as independent risk-enhancing factors into established clinical risk stratification algorithms, such as the classic 10-year ASCVD (atherosclerotic cardiovascular disease) risk score or the European SCORE2 model (117). This integration holds the potential to significantly enhance predictive accuracy, particularly for patients classified within the intermediate or borderline risk strata. Indeed, patient-specific hemodynamic shear stress profiling has been demonstrated to partially elucidate the susceptibility to atherosclerosis in patients devoid of standard modifiable cardiovascular risk factors (SMuRF-less), offering profound potential to guide targeted prophylactic interventions (118). Future investigations must leverage large-scale prospective clinical trials to validate whether such composite risk models—integrating hemodynamic parameters, established clinical scores (ASCVD/SCORE2), and multi-omics biomarkers—demonstrate significant superiority over conventional risk stratification strategies, ultimately driving the paradigm shift from “one-size-fits-all” to “tailor-made” precision prevention and management of atherosclerosis.

## 8 Limitations and critical assessment of existing research

While this review has systematically delineated the mechanobiological mechanisms by which shear stress drives atherosclerosis, it is imperative to objectively acknowledge the inherent methodological and translational limitations of the currently cited literature. First, concerning *in vitro* mechanistic studies, the majority of the molecular pathway explorations cited herein (e.g., the activation mechanisms of Piezo1 and cGAS-STING) rely heavily on two-dimensional (2D) parallel-plate flow chambers or cone-and-plate viscometer models. Although these conventional *in vitro* systems offer precise control over the application of specific fluid shear stress, they fail to authentically recapitulate the complex three-dimensional (3D) microenvironment of the human arterial wall (119). Specifically, these models typically lack multicellular co-culture and paracrine crosstalk among vascular endothelial cells, smooth muscle cells, and fibroblasts; furthermore, they fail to integrate the multiphysics coupling effects of pulsatile wall tension (circumferential strain) and fluid shear stress. This deficiency may lead to the overestimation of certain “irreversible” phenotypes (such as profound cellular senescence) observed *in vitro* when compared to the complex compensatory networks within the actual human body (120).

Second, at the level of *in vivo* animal experiments, a substantial proportion of studies investigating the *in vivo* pathogenicity of OSS depend on rodent models (such as ApoE-/- or LDLR-/- mice combined with partial carotid artery ligation models) (121). While this model effectively induces localized hemodynamic disturbances and accelerates plaque formation, researchers must remain vigilant regarding the significant hemodynamic disparities between species. The murine heart rate (approximately 500–600 beats/min) and basal wall shear stress magnitude are substantially higher than those in humans, and the induced plaques often fail to fully replicate the complex histological landscape of human vulnerable plaques (e.g., fibrous cap thickness, necrotic core proportion, and rupture propensity) (4). Therefore, considerable caution must be exercised when directly extrapolating mechanotransduction pathways (such as EndoMT or specific epigenetic modifications) validated in these models to human carotid or coronary artery lesions.

Finally, regarding the translation of clinical and imaging evidence, although multimodal imaging combined with CFD technology has made significant strides in quantifying shear stress, the currently cited studies supporting the clinical prognostic value of wall shear stress are predominantly limited to single-center, retrospective analyses, or observational cohorts with small sample sizes (122). This inevitably introduces selection bias. Crucially, there remains a glaring paucity of large-scale, multicenter, prospective randomized controlled trials (RCTs) to substantiate whether intervention decisions based on shear stress quantification (e.g., WSS-guided optimization of stent implantation) can genuinely improve long-term hard cardiovascular outcomes in patients (123). Although multimodal quantification represents a highly promising evaluation strategy at present, the level of evidence in the existing clinical literature remains tenuous prior to its widespread translation into routine clinical practice.

## Data Availability

The original contributions presented in the study are included in the article/Supplementary Material, further inquiries can be directed to the corresponding author.
